# Glanders and Its Suppression; Experiments with Mallein

**Published:** 1899-01

**Authors:** J. M. Wright

**Affiliations:** Professor of Pathology and Contagious Diseases, M’killip Veterinary College; Assistant State Veterinarian of Illinois


### GLANDERS AND ITS SUPPRESSION; EXPERIMENTS WITH MALLEIN.[1]

By J. M. Wright, M.D.C.,
PROFESSOR OF PATHOLOGY AND CONTAGIOUS DISEASES, M'KILLIP VETERINARY COLLEGE; ASSISTANT STATE VETERINARIAN OF ILLINOIS.

Glanders is one of the oldest diseases on record to which the equine race is heir. We find descriptions of it given over twenty-three hundred years ago. While it is one of the oldest diseases, it

[1] Delivered before the Interstate Association of Livestock Sanitary Boards, Omaha, Nebraska, October 11, 1898.

is the most loathsome, contagious, and dreaded known to generations, past and present.

It is essentially a disease of the equine race, but by inoculation can be transmitted to other animals—guinea-pigs, field-mice, and even men. Once introduced into a province, State, or country it remains, notwithstanding the efforts of the ablest men of the ages. It exists to-day in every part of the world inhabited by the horse, mule, or ass. It causes greater destruction in warm countries than in cold, even with the same management and care of the animals. All countries, in all ages, past and present, have made determined efforts to get rid of the scourge. There have been repeated attempts, from the beginning down to the present time, to cure animals suffering from the malady, by good management and the application of medicines.

The French, at the beginning of the present century, came to the conclusion that it was not contagious, and repealed all police sanitary laws and attempted to stamp it out by the use of medicine. Note what followed: In less than twenty-five years there was a most deplorable condition of affairs in every part of the French republic. The percentage of mortality increased from a minimum to a high rate per cent. The destruction of property and the loss of human life became so great that, before many years had passed, they established new police sanitary laws, and enforced them with greater vigilance than ever before. This had the desired effect of lessening the percentage of mortality.

I personally know of numerous instances where effort has been made to effect cures by aid of therapeutical agents in this country; one instance where barns were secured outside the city limits and many glandered patients taken for treatment. Such efforts were ineffectual, as they never returned a single horse cured. Horses had been treated in another barn for two years, until, as a State officer, I discovered them and what was going on. I took possession of the premises. The owner had never owned more than four horses at any one time, but during the period that they were treated he lost eleven diseased with glanders. When I took possession of the place I found three horses in the barn—a large sorrel horse, so badly diseased with farcy and glanders that he could scarcely walk; a large bay horse, almost as badly diseased; and a gray horse, which had been there for years, in good condition—at first sight would be regarded as in a perfect state of health; but upon closer examination he was found to have glanders in the chronic form. It is needless to say that I destroyed them all.

I could relate many similar instances just as provoking as the above, but time will not permit.

Attempts have been made in the West to effect a cure by turning glandered horses out on the ranges. If this were done in Colorado, Wyoming, Montana, and Dakota when the weather is fine, in the summer months, the animals would gain in flesh, and many of the symptoms would become modified or disappear; but when feed becomes scanty in the fall, and cold rains, sleet and snow, and blizzards with their chilly blasts come, the disease would assume the acute form and the animals die in great numbers. Some of the stronger ones would, perhaps, live through the winter, and with the return of spring, its fine weather and plenty of food, improve and continue in the chronic form, spreading the seeds of contagion.

Nasal discharge and farcy are only manifestations of generalized or systemic glanders in 90 per cent. of animals afflicted with the disease. All such animals should be destroyed without question. It is not the animals that have the disease in the above form which are to be most dreaded, for they are usually destroyed as soon as discovered, but it is the animals having it in the occult form which spread the seeds of contagion in a community when it is once planted there. Some will remain in apparent health for months and years, and have only slight periodical discharges from the nostrils, and often so slight as to escape notice. Others will not even have such discharge, but continue in apparently perfect health for months and years, or until some debilitating influence is brought to bear upon them. Should they suffer from exposure, be poorly fed, or be attacked with catarrh, influenza, pleurisy, or pneumonia, it would precipitate the disease into an acute form very quickly.

It behooves us to detect all animals which have been exposed and are infected in an incipient form. As this cannot be done by physical examination, we must cast about for some element or agent to aid us. Aided by mallein, and following its use by postmortems, I have found that on an average 40 per cent. of all animals exposed are actually infected with glanders in its incipient form, with absolutely no external manifestations of the disease; in 95 per cent. the lesions will be found in the lungs, liver, or mesenteric lymphatics, or in all three of them; in not more than 1 per cent. will there be lesions in the upper air-passages or in the larger bronchial tubes.

The modes of invasion are many, and these vary according to circumstances and condition of the animals, the state of the sanitary surroundings, the climate, and the season of the year. An animal may become infected by rubbing his nose against the nose of one with a profuse discharge, or in like manner from the walls of the stall, feed-troughs, mangers, etc. It can be produced by rubbing the contents of a glanderous ulcer, tubercle, or the discharge from the nostrils on a mucous membrane or on the skin where there are no lesions, but if it be applied to the latter it must be well rubbed in. Infection may take place in the mouth and alimentary canal, through wounds or abrasions, when obtained from bridle-bits, hitching-posts, walls of the stall, manger, feed-troughs, or from infected feed, such as grain, hay, or straw. I feel safe, however, in saying that less than 10 per cent. of all cases of infection take place in the above-described manner. When it does occur, there will invariably be, first, a localized lesion, which will spread by means of the lymphatics into the surrounding parts, extending further and further until it becomes general. The common watering-trough is the place where animals become infected in a majority of cases. A horse is watered one or more times each day, when his stomach is empty and he is thirsty. Usually under such circumstances he will ingest large quantities, which pass at once from the stomach into the intestinal tract, where it is absorbed almost immediately by two sets of absorbents: First, the portal circulation, which carries this infected matter and pours it into the liver, where it for the first time has to pass through the capillary system, and where the bacilli have their first opportunity to escape from the vessels and gain entrance into the tissue-spaces and lymphatic vessels, where they produce their characteristic lesions. Those which fail to escape are emptied into the posterior vena cava and the next capillary system they pass through is in the lungs, where they again have the same opportunity as when passing through the liver. Secondly, the mesenteric lymphatic system, which collects and pours its contents into the anterior vena cava through the thoracic duct. The infected material, having thus found its way into the venous circulation, quickly finds its way into the lungs, where it for the first time passes through the capillary system, and the bacilli have an opportunity to escape into the tissue-spaces. It will be noticed that the early primary lesions are in the parenchyma of the lungs and not in the bronchial tubes, which explodes the inhalation theory.

Farcy may be produced by cutaneous infection in various ways, such as wounds, abrasions, hypodermatically, and by glanderous material being placed upon the skin or mucous membrane. If on the skin it will be necessary to apply friction or to rub it thoroughly into the openings of the same. Ninety per cent. of all cases of farcy are produced by the bacilli escaping from the lungs or through them, gaining entrance into the general arterial circulation, and carried to the extremity of the artery, into the capillary system, where the bacilli escape from these small vessels into the small tissue-spaces and smaller lymphatics. When they have once gained entrance into these tissue-spaces they are permitted to remain at rest for a longer or shorter period of time, thus giving them ample opportunity (if not destroyed by nature's elements) to multiply and produce their characteristic lesions. And if such lodgement and development should be near the surface of the body there would be manifest farcy-buds. I am supported in this statement by the fact that farcy-buds may appear on different parts of the body almost simultaneously, which would not take place if they were produced by cutaneous infection.

(To be continued.)

